# Editorial: Mechanisms, Measurement, and Management of Vasogenic Edema After Stroke

**DOI:** 10.3389/fneur.2022.865078

**Published:** 2022-02-24

**Authors:** Gabriel Broocks, Jens Minnerup, Shervin Kamalian, Andre Kemmling

**Affiliations:** ^1^Department of Diagnostic and Interventional Neuroradiology, University Medical Center Hamburg-Eppendorf, Hamburg, Germany; ^2^Department of Neurology With Institute of Translational Neurology, University Hospital Münster, Münster, Germany; ^3^Department of Neuroradiology, Harvard Medical School and Massachusetts General Hospital, Boston, MA, United States; ^4^Department of Neuroradiology, University Marburg, Marburg, Germany

**Keywords:** stroke, edema, ischemia, imaging, infarction

## Introduction

Cerebral tissue edema is a pathophysiological hallmark of acute ischemic injury. Cerebral ischemia induced by critical brain perfusion results in disturbed water homeostasis, and subsequently a net uptake of water and cations into the ischemic tissue. In large ischemic lesions, as a severe complication, progressive water uptake may lead to severe space-occupying edema within the first days after stroke onset with mortality up to 80% (1, 2). Therefore, the identification and validation of reliable neuroimaging biomarkers for developing brain edema remains an elementary challenge of stroke imaging despite recent advances in artificial intelligence-based automated imaging tools. Furthermore, it has recently been observed that treatment strategies in ischemic stroke directly affect the formation of vasogenic edema following stroke (3–5). Hence, ischemic vasogenic edema may be a therapeutic target for both endovascular treatment, as well as novel adjuvant neuroprotective drugs (6). The aim of this Research Topic was to investigate neuroimaging biomarkers of ischemic vasogenic edema in acute stroke for imaging triage, measurement of treatment effects, and prediction of functional outcome.

## Mechanisms of Vasogenic Edema After Stroke

The identification of variables associated with the progression of ischemic edema is a fundamental challenge in the asssessment of acute ischemic stroke patients on admission, particularly considering modifiable variables that could be used to alter the progression of infarction. Nawabi et al. hypothesized that baseline serum glucose, which is known to be associated with functional outcome and response to endovascular treatment, interacts with the effect of tissue water uptake on the occurrence of intracerebral hemorrhage (ICH), a major complication after reperfusion therapy. It was observed that a higher degree of early tissue water uptake and high admission serum glucose were both independent predictors of ICH. Although a clear causal relationship remains speculative, stricter glucose control, and monitoring could be tested to reduce the risk of ICH in patients undergoing thrombectomy.

In the further course of ischemic brain infarct, vasogenic edema progresses and constitutes up to 35% of the total infarct lesion volume. Konduri et al. sought to elucidate the role of edema in subacute lesion progression and its influence on unfavorable functional outcome by quantifying net water uptake including patients from the MR CLEAN trial. It was observed that edema volume ususally increases in the period between 1 day and 1 week after acute ischemic stroke, depending on the degree of recanalization, and was significantly associated with functional outcome.

So far, limited data exist regarding the hemispheric differences in the incidence of stroke-related complications and outcomes of patients with extensive baseline stroke. Li et al. assessed the hemispheric differences with characteristics, complications, and outcomes of patients suffering from large hemisphere infarctions (LHI). Li et al. observed that right-sided patients with LHI had higher frequency of atrial fibrillation and cardio-embolism than the left-sided patients, whereas stroke lateralization was not an independent predictor of mortality and unfavorable outcome.

## Measurement of Vasogenic Edema After Stroke

Malignant cerebral edema (MCE) following ischemic stroke regularly results in very poor outcomes. Hence, the early prediction of MCE might help to identify patients that could benefit from decompressive surgery. Quantitative net water uptake (NWU) has been described as a predictor of MCE; however, the utilization of CT perfusion and lesion segmentation is currently necessary for NWU assessment. Fu et al. proposed a new Image Patch-based Net Water Uptake (IP-NWU) procedure that only uses non-enhanced admission CT and does not need lesion segmentation. In summary, IP-NWU showed a high diagnostic accuracy to predict MCE, with an AUC of 0.86. By combining IP-NWU with imaging features through a random forest classifier, the radiomics model achieved even an AUC of 0.96. Xu et al. aimed to investigate whether NWU calculated in standardized and blindly outlined regions of the middle cerebral artery territory can predict the development of MCE, aiming to further standardize and simplify the procedure of NWU quantification. Xu et al. concluded that NWU calculated in standardized and blindly outlined regions of the middle cerebral artery territory was a good predictor for the development of MCE (AUC: 0.73).

Steffen et al. performed a pilot study to investigate the potential value of dual-energy dual-layer CT after mechanical thrombectomy (MT) for the improved assessment of ischemic edema, as it is known that contrast staining after MT might directly affect the measurement of NWU (7). NWU was measured in conventional polychromatic CT images and virtual non-contrast images in a blinded fashion. It was observed that NWU may be quantified precisely on conventional CT images, as the underestimation of ischemic edema due to contrast staining was low. Nonetheless, a relevant proportion of patients after MT (2 out of 10 patients) might show significant contrast staining resulting in edema underestimation.

The measurement of quantitative NWU is not yet established in acute stroke triage. Bechstein et al. propose a simple imaging score to be applied on baseline non-enhanced CT images (i.e., “NEMMI score”). It was hypothesized that this score, consisting of measurements of visually evident ischemic changes (sum of the maximum diameter anterior-poster + mediolateral of the hypoattenuated lesion in baseline-CT multiplied by a hypoattenuation factor) on non-enhanced CT predicts malignant middle cerebral artery infarctions with a high diagnostic power. In conclusion, the authors observed that the NEMMI score might serve as a quick and simple rating tool of early ischemic changes on CT and could serve as a surrogate marker for developing malignant edema. Its diagnostic accuracy was similar to CTP and clinical parameters.

Finally, the quantification of early edematous water uptake might have particular importance in the assessment of patients with extensive baseline infarction (i.e., low ASPECTS). The rating of ASPECTS is based on the presence of hypoattenuation of the ischemic brain tissue, which is directly related to early infarct, and the degree of relative hypoattenuation can be used to quantify ischemic edema on CT. The purpose of the pilot study of Broocks et al. was to evaluate how early tissue water uptake in acute ischemic brain might determine lesion fate and functional outcome in low ASPECTS patients undergoing MT. In this single center analysis of 155 low ASPECTS patients, NWU showed to be an independent predictor of outcome. While low NWU was a beneficial constellation regarding the effect of recanalization on outcome, high NWU was associated with futile recanalization. Further research is necessary to elucidate the value of quantitative NWU in the assessment of low ASPECTS patients.

## Management of Vasogenic Edema After Stroke

Currently it is tested, whether Glibenclamide, a sulfonylurea, improves outcome in large hemispheric stroke and reduces formation of ischemic edema (6). Zhao et al. aim to explore the efficacy of small doses of oral glibenclamide in perihematomal edema (PHE) and the prognosis of patients with ICH in a clinical trial (GATE-ICH, NCT03741530). The study assesses the effects of small doses of oral glibenclamide in reducing the PHE after ICH and improving the 90-day prognosis of patients. Similarly, Jingjing et al. assessed whether sulfonylureas are associated with lower PHE and improved clinical outcomes in diabetic patients who have acute basal ganglia hemorrhage. In this case-control study, Jingjing et al. concluded that pretreatment with sulfonylureas were associated with lower PHE and relative PHE on admission. No significant effect was found on the clinical outcomes when the patients were discharged. Still, the authors see a need for further studies to assess the potential clinical benefit using sulfonylureas for ICH patients.

## Discussion and Future Challenges

Neuroimaging biomarkers of ischemic edema in acute stroke are highly relevant for imaging triage of stroke patients, to measure and compare treatment effects, and to predict functional outcome. First, it is important to realize that the term “edema” is regularly connected with different meanings, such as “swelling” (i.e., expansion of an infarct lesion with adjacent anatomy shift), or “net uptake of water” into the ischemic tissue which is evident by progressive hypoattenuation even below visually evident brain swelling. The precise quantification of early edema remains an elementary challenge, and NWU may particularly serve as a tool to select patients for treatment. Current trials may have missed this opportunity.

For instance, it is yet uncertain how to select patients with a low ASPECTS for endovascular treatment (8, 9). The currently active low ASPECTS trials apply different inclusion criteria based on ischemic core volume or ASPECTS. However, the degree of hypoattenuation, which directly reflects net uptake of water, is currently not considered as imaging biomarker in stroke triage. As described in the above-mentioned pilot study, the degree of water uptake as quantified on baseline CT might serve as a predictor of outcome, and could be tested as a tool to select low ASPECTS patients for thrombectomy (10). A further application of edema imaging is to guide adjuvant treatment. The CHARM trial (NCT02864953) is a phase 3 study to evaluate the efficacy and safety of intravenous glibenclamide for severe cerebral edema. However, this study only includes patients with visually evident and apparent large infarct lesions, in particular patients after thrombectomy (likely the majority or large minority of patients) when inclusion criteria are met on follow-up MRI DWI scans. Alternatively, NWU on baseline CT could be used to select patients for glibenclamide administration early prior to visually apparent brain swelling, before, or during thrombectomy to mediate the detrimental effect of net water uptake at an early ischemic lesion stage, which may progress to high levels quickly often resulting in malignant edema and futile recanalization (3, 11, 12).

## Author Contributions

All authors listed have made a substantial, direct, and intellectual contribution to the work and approved it for publication.

## Conflict of Interest

The authors declare that the research was conducted in the absence of any commercial or financial relationships that could be construed as a potential conflict of interest.

## Publisher's Note

All claims expressed in this article are solely those of the authors and do not necessarily represent those of their affiliated organizations, or those of the publisher, the editors and the reviewers. Any product that may be evaluated in this article, or claim that may be made by its manufacturer, is not guaranteed or endorsed by the publisher.
